# Characterization of antimicrobial susceptibility, extended-spectrum β-lactamase genes and phylogenetic groups of Shigatoxin producing *Escherichia coli* isolated from patients with diarrhea in Iran

**DOI:** 10.1186/s12941-021-00430-1

**Published:** 2021-04-15

**Authors:** Erfaneh Jafari, Mana Oloomi, Saeid Bouzari

**Affiliations:** 1 Molecular Biology Department, Pasteur Institute of Iran, Tehran, Iran; 2 National Escherichia Coli Reference Laboratory, Pasteur Institute of Iran, Tehran, Iran

**Keywords:** STEC, Multi-drug resistance, Extended-spectrum β-lactamase, Phylogenetic groups, MLST

## Abstract

**Background:**

Shiga toxin‐producing *Escherichia coli* (STEC) are among common foodborne bacterial pathogens and healthy livestock are the main source of this bacterium. Severe diseases attribute to two types of cytotoxin Stx1 and Stx2, which are also called Shiga toxin (Stx). Infection of humans with STEC may result in Acute diarrhea with or without bleeding, hemorrhagic colitis (HC) and the hemolytic uremic syndrome (HUS). As antibiotic resistance is increasingly being reported among STEC isolates obtained from livestock and patients worldwide, in this study the pattern of antibiotic resistance in clinical isolates was determined.

**Methods:**

Stool samples were collected from patients with diarrhea. All samples were cultured and identified by biochemical and molecular tests. Antimicrobial susceptibility test and assessment of extended-spectrum β-lactamase (ESBL)-related genes were conducted. Moreover, phylogenetic groups were analyzed using quadruplex PCR, and DNA analysis assessed multi-locus sequence types (MLST).

**Results:**

Out of 340 *E. coli* samples, 174 were identified as STEC by PCR. Antimicrobial susceptibility test results showed that, 99.4%, 96% and 93.1% of isolates were susceptible to imipenem/ertapenem, piperacillin–tazobactam and amikacin, respectively. The highest resistance was towards ampicillin (68.4%), followed by trimethoprim–sulfamethoxazole (59.8%), and tetracycline (57.5%). A total of 106 (60.9%) isolates were multidrug resistance (MDR) and 40.8% of isolates were determined to be extended spectrum β-lactamase producers. In 94.4% of isolates, genes responsible for ESBL production could be detected, and *blaTEM* was the most prevalent, followed by *blaCTX-M9*. Furthermore, phylogenetic grouping revealed that majority of STEC strains belonged to Group C, followed by Groups E, B2 and A. MLST unveiled diverse ST types.

**Conclusion:**

A periodical surveillance studies and thorough understanding of antibiotic resistant profiles in STEC isolates could help select effective antibiotic treatment for patients and develop strategies to effectively manage food contamination and human infections.

## Background

Shiga toxin‐producing *Escherichia coli* (STEC), including O157:H7 and non-O157 serotypes, are among common foodborne pathogens. Outbreaks of several human gastrointestinal diseases, including acute diarrhea with or without bleeding, hemorrhagic colitis (HC) and the hemolytic uremic syndrome (HUS), are caused by STEC [1, 2]. The elderly and children are more plausible to serious complications caused by this bacterium [2]. Healthy livestock, especially cattle, are the main reservoir of STEC and human infection is often due to contamination of food or water resources [2, 3]. Human virulent STEC produce two types of Shiga toxin (Stx), Stx1 and Stx2 which each have different variants. This bacterium may also contain other virulence factors such as intimin (*eae*), that is involved in creating attaching and effacing (A/E) lesions [2, 3]. In cases attributed to STEC, antibiotics should not be administrated as they may lead to bacterial lysis and raised production of Stx and also increased number of antibiotic‐resistant strains [2].

In recent years, due to extensive application of various antibiotics (such as β-lactams) against infections, high levels of antibiotic resistance and extended-spectrum beta-lactamase (ESBL)-producing bacteria are being detected [4]. Extended-spectrum beta-lactamases are specific enzymes produced by *Enterobacteriaceae.* Their common spread mechanism is by horizontal gene transfer and can be bacterial chromosome and plasmid mediated [5]. The main groups of ESBL enzymes include Class A, C or D enzymes which are generally inhibited by clavulanic acid or tazobactam. The plasmid mediated Class A β-lactamase, including TEM, SHV, and CTX-M, and chromosomally mediated Class D β-lactamase OXA are the most predominant among *Enterobacteriaceae* [6]. The plasmids that contain the ESBL genes are also often encoded with different classes of antibiotics [7], which lead to the development of multidrug resistant (MDR) strains.

*Escherichia coli* clinical strains have been classified into several phylogenetic groups (A, B1, B2, C, D, E, and F), based on the combination of certain genes and a DNA fragment (*chuA, yjaA, arpA* genes and TspE4.C2) [8]. Evaluation of the relationship between pathogenicity and phylogeny also demonstrated that the strains belonging to different phylogroups are associated with the source of isolation [8]. The commensal *E. coli* strains usually belong to groups A and B1 [9], whereas virulent extra-intestinal strains mainly belong to groups B2 and to a lesser extent, to group D [10]. Phylogenetic characterization of *E. coli* clinical strains provides information about the relation of strains and disease and frequency of their occurrence in the environment. But compared to Uropathogenic *E. coli,* few studies have determined phylogenetic groups of diarrheagenic *E. coli*. Also the Clermont triplex PCR method, which is only capable of determining phylogroups A, B1, B2, and D, has been used more extensively. Therefore, as disposition of phylogroups for diarrheagenic *E. coli* is still unclear, STEC could belong to any of the phylogroups [11].

As antibiotic resistance is increasingly being reported among STEC isolates obtained from livestock and patients worldwide, and death is specifically associated to antibiotic‐resistant STEC strains, in this study the pattern of antibiotic resistance, as well as ESBL production and phylogenetic groups associated with clinical isolates, was determined.

## Materials and methods

### Sampling and processing

During 1-year period (2014), diarrhea patients’ stool samples inoculated on MacConkey agar were collected from different reference hospitals. Following standard microbiological techniques [12], 340 samples from patients ages between 5 months to 92 years old had been biochemically confirmed as *E. coli*. Five colonies from each of the confirmed isolates’ MacConkey agar plate tested by PCR to identify the presence or absence of *stx1A*, *stx2A*, and *eae* genes [13, 14]. *Escherichia coli* strain O157/H7 was used as a positive control. Non-pathogenic *E. coli* strain DH5α was used as negative control to monitor PCR contamination. All *E. coli* isolates were submitted to DNA extraction by boiling method as follows: 1 ml from overnight cultures of each colony was centrifuged at 12000 rpm for 2 min. The supernatant was discarded, and the pellet was washed with 1 ml of normal saline by centrifugation at 12000 rpm for 1 min. The pellet was resuspended in 200 μl DNase/RNase free distilled water (ThermoScientific) and subjected to boiling at 100 °C for 10 min. After centrifugation at 14000 rpm for 2 min, 50 μl of supernatant was collected to be used as template. The detection of virulence genes were carried out under the following PCR conditions: 95 °C for 3 min, 30 cycles of 94 °C for 1 min, 60 °C for 45 s, 72 °C for 45 s, and a final extension at 72 °C for 5 min.

### Antimicrobial susceptibility testing

Antimicrobial susceptibility tests were performed on Mueller–Hinton agar (Himedia, India) using commercial antimicrobial discs (BD BBL, USA), based on standard disk diffusion method and according to the guidelines of the clinical and laboratory standards institute (CLSI) [15]. The antibiotic discs were ampicillin (10 μg), amikacin (30 μg), ceftazidime (30 μg), cefotaxime (30 μg), ciprofloxacin (5 μg), ertapenem (10 μg), imipenem (10 μg), levofloxacin (5 μg), piperacillin–tazobactam (100 μg + 10 μg), tetracycline (30 μg) and trimethoprim–sulfamethoxazole (1.25 μg + 23.75 μg). Multidrug resistance was defined by discerning non-susceptibility to at least 1 antibiotic in ≥ 3 antimicrobial categories [16]. *Escherichia coli* ATCC 25922 was used for quality control. Detection of ESBLs was performed by combined disc assay [15], using ceftazidime (30 μg) and cefotaxime (30 μg) discs, alone and in combination with clavulanic acid (10 μg). The production of ESBL was determined by the expansion of ≥ 5 mm of the zone diameters of combined discs compared to ceftazidime and cefotaxime zones.

### Molecular identification of ESBL producing strains

PCR was performed to detect β-lactamase genes *blaCTX-M9*, *blaSHV*, *blaOXA* and *blaTEM* using primer sequences presented in Table 1 [17–20]. The PCR procedure was carried out in a total volume of 25 μl, using 12 μl of Taq DNA Polymerase Mix Red-Mgcl_2_ 2 mM (Ampliqon), 9 μl of DNase/RNase free distilled water (ThermoScientific), 1 μl of 10 pM for reverse and forward primers, and 2 μl of DNA template.

**Table 1:** Primer sequences and sizes of PCR products

Target GENE	Amplicon size (bp)	Primer Sequence (5′ to 3′)	References
Virulence genes			
*Stx1A*	244	F: CGATGTTACGGTTTGTTACTGTGACAGC	[13]
		R: AATGCCACGCTTCCCAGAATTG	
*Stx2A*	324	F: GTTTTGACCATCTTCGTCTGATTATTGAG	[13]
		R: AGCGTAAGGCTTCTGCTGTGAC	
*eae*	570	F: AGGCTTCGTCACAGTTG	[14]
		R: CCATCGTCACCAGAGGA	
β-lactamase genes			
*blaCTX-M9*	856	F: GTGACAAAGAGAGTGCAACGG	[17]
		R: ATGATTCTCGCCGCTGAAGCC	
*blaSHV*	768	F: TCGCCTGTGTATTATCTCCC	[18]
		R: CGCAGATAAATCACCACAATG	
*blaOXA*	438	F: GCGTGGTTAAGGATGAACAC	[19]
		R: CATCAAGTTCAACCCAACCG	
*blaTEM*	963	F: GCGGAACCCCTATTTG	[20]
		R: ACCAATGCTTAATCAGTGAG	
Phylogenetic genes			
*chuA*^a^	288	F: ATGGTACCGGACGAACCAAC	[8]
		R: TGCCGCCAGTACCAAAGACA	
*yjaA*^a^	211	F: CAAACGTGAAGTGTCAGGAG	[8]
		R: AATGCGTTCCTCAACCTGTG	
*arpA*^a^	400	F: AACGCTATTCGCCAGCTTGC	[8]
		R: TCTCCCCATACCGTACGCTA	
TspE4.C2^a^	152	F: CACTATTCGTAAGGTCATCC	[8]
		R: AGTTTATCGCTGCGGGTCGC	
*arpA*__gpE_	301	F: GATTCCATCTTGTCAAAATATGCC	[8]
		R: GAAAAGAAAAAGAATTCCCAAGAG	
*trpA*__gpC_	219	F: AGTTTTATGCCCAGTGCGAG	[8]
		R: TCTGCGCCGGTCACGCCC	
*trpA*__Internal control_	489	F: CGGCGATAAAGACATCTTCAC	[8]
		R: GCAACGCGGCCTGGCGGAAG	

^a^Quadruplex

### Identification of phylogroups

All STEC strains were appointed to a phylogenetic group (A, B1, B2, C, D, E or F) based on Clermont’s quadruplex multiplex PCR scheme [8]. Genomic DNA of the isolates was used. Instead of the DNA template, water was used as negative control. Agarose gel electrophoresis of the PCR product was carried out in 2% agarose gel containing DNA Gel dye.

### Multi-locus sequence typing (MLST)

Although MLST is one of the choice methods for typing of epidemiologically important strains, but as it is a costly and time-consuming technique, only a fraction of the *E. coli* pathogenic isolates were selected to perform the test. Pasteur MLST system was carried out by amplifying eight house-keeping genes (dinB, icdA, pabB, polB, putP, trpA, trpB,and uidA) from *E. coli* chromosomal DNA. The amplified products were purified and subjected to Sanger dideoxy DNA sequencing (Microsynth, Switzerland). To determine sequence types (STs), the sequences of these genes were then compared with known alleles at each locus, which are available from Institute Pasteur’s MLST website, at www.pasteur.fr/mlst.

### Data analysis

Comparison of proportions and graphic were performed with IBM SPSS 19.0 software and Microsoft Excel (Microsoft Cooperation, 2010). Pearson Chi-square test was used to determine the difference in resistance between ESBL positive and ESBL negative isolates. A two-sided *P*-values < 0.05 were considered statistically significant. Descriptive statistics such as frequency and percentage were also used to indicate categorical data.

## Results

### Sample characteristics

In total, 340 *E. coli* isolates were tested for *stx1A, stx2A* and *eae* genes using PCR; from which STEC’s virulence genes were detected in 174 (51.2%) of diarrhea cases. Frequency of virulence factors among confirmed samples was as follows; stx1 171 (98.3%), stx2 10 (5.7%), eae 16 (9.2%), and five different virulence profiles were observed i.e., 148 (85.0%) isolates showed only *stx1* gene, 15 (8.6%) were positive for *stx1* and *eae,* 7 (4.0%) for *stx1* and *stx2,* 3 (1.7%) for *stx2* and only one of the isolates was positive for all 3 genes. Moreover, the positive cases comprised of 97 males and 77 females with male: female ratio of 1.26:1; from which 44 male and 27 female belonged to children under 6 years, 27 male and 25 female belonged to 10–30 year olds, and the rest of the isolates were from patients older than 30 years (Table 2).

**Table 2:** Demographic characteristic of patients

Variables		All Samples (n = 340)	STEC (n = 174)	MDR (n = 106)	ESBL + (n = 71)
Gender No. (%)	Male	184 (54.1)	97 (55.7)	61 (57.5)	40 (56.3)
	Female	156 (45.9)	77 (44.3)	45 (42.5)	31 (43.7)
Age No. (%)	≤ 6 year	126 (37.1)	71 (40.8)	43 (40.6)	26 (36.6)
	7–30 year	105 (30.9)	52 (29.9)	31 (29.2)	25 (35.2)
	> 30 year	109 (32.0)	51 (29.3)	32 (30.2)	20 (28.2)

*STEC* Shiga toxin‐producing *Escherichia coli*, *MDR* multidrug resistant, *ESBL* extended spectrum β-lactamase

### Antibiotic resistance profile

Based on the evaluation of antibiotic susceptibility pattern against 11 antimicrobial agents (Table 3), among the 174 STEC isolates, 20.1% were sensitive to all tested antibiotics, and 79.9% showed resistance to at least one antibiotic. From these isolates, 60.9% exhibited MDR profile and 40.8% were ESBL producers. The resistance to ampicillin, trimethoprim–sulfamethoxazole and tetracycline was highly prevalent (68.4%, 59.8% and 57.5%, respectively); whereas resistance rate to cefotaxime, ceftazidime, ciprofloxacin and levofloxacin were lower than 45%. Sensitivity toward imipenem and ertapenem, were 99.4% and more than 90% of isolates were also susceptible to piperacillin–tazobactam, and amikacin. All ESBL producing isolates had high rates of resistance toward cefotaxime and ampicillin, while being susceptible to imipenem and ertapenem. Significantly, the rates of resistance to cefotaxime (98.6% vs. 6.8%), ceftazidime (62.0% vs. 4.9%), levofloxacin (45.1% vs. 9.7%), and ciprofloxacin (42.4% vs. 13.6%) were higher in ESBL + isolates than in ESBL- isolates.

**Table 3:** Antimicrobial susceptibility patterns of STEC isolates

Antibiotic	Total (n = 174)			ESBL– (n = 103)			ESBL + (n = 71)			*P*-value
	R No. (%)	I No. (%)	S No. (%)	R No. (%)	I No. (%)	S No. (%)	R No. (%)	I No. (%)	S No. (%)	
Cefotaxime	77 (44.3)	3 (1.7)	94 (54.0)	7 (6.8)	2 (1.9)	94 (91.3)	70 (98.6)	1 (1.4)	–	< 0.001
Ceftazidime	49 (28.2)	20 (11.5)	105 (60.3)	5 (4.9)	3 (2.9)	95 (92.2)	44 (62.0)	17 (23.9)	10 (14.1)	< 0.001
Piperacillin-tazo	1 (0.6)	11 (6.3)	162 (93.1)	–	5 (4.9)	98 (95.1)	1 (1.4)	6 (8.2)	64 (90.1)	0.298
Ertapenem	1 (0.6)	–	173 (99.4)	1 (1.0)	–	102 (99.0)	–	–	71 (100)	0.405
Imipenem	–	1 (0.6)	173 (99.4)	–	1 (1.0)	102 (99.0)	–	–	71 (100)	0.405
Ciprofloxacin	44 (25.3)	10 (5.74)	120 (69.0)	14 (13.6)	4 (3.9)	85 (82.5)	30 (42.4)	6 (8.2)	35 (49.4)	< 0.001
Levofloxacin	42 (24.1)	3 (1.7)	129 (74.2)	10 (9.7)	2 (1.94)	91 (88. 4)	32 (45.1)	1 (1.4)	38 (53.5)	< 0.001
Amikacin	1 (0.6)	6 (3.4)	167 (96.0)	–	3 (2.9)	100 (97.1)	1 (1.4)	3 (4.2)	67 (94.4)	0.429
Tetracyclines	100 (57.5)	4 (2.3)	70 (40.2)	44 (42.7)	3 (2.9)	56 (54.4)	56 (78.9)	1 (1.4)	14 (19.7)	< 0.001
Ampicillin	119 (68.4)	5 (2.9)	50 (28.7)	49 (47.6)	5 (4.9)	49 (47.6)	70 (98.6)	–	1 (1.4)	< 0.001
Trimethoprim-sulfa	104 (59.8)	–	70 (40.2)	43 (41.7)	–	60 (58.3)	61 (85.9)	–	10 (14.1)	< 0.001

*R* resistant, *I* intermediate, *S* sensitive, *ESBL* extended spectrum β-lactamase, *STEC* Shiga toxin‐producing *Escherichia coli*

A total of 106 isolates were considered as multidrug resistance, from which 43 (40.6%), 36 (33.9%) and 27 (25.5%) belonged to 3, 4 and 5 classes of antibiotics, respectively. The isolates resistance pattern ranged from 3 to 7 antibiotics and showed 20 different profiles that are shown in Table 4. Among MDR isolates, 40.6% belonged to children ≤ 6 year, and 64.2% were ESBL producers. The frequency of 3, 4 and 5 antibiotic groups in MDR ESBL positive strains was 13 (19.1%), 29 (42.7%) and 26 (38.2%), respectively.

**Table 4:** Antibiotic resistance profile in 106 MDR isolates

Antibiotic profile	No. of antibiotic groups	No. (%)
CTX/CAZ/CIP/LVX/Te/AM/SXT	5	19 (17.9)
CTX/CIP/LVX/Te/AM/SXT	5	8 (7.6)
CTX/CAZ/CIP/LVX/AM/SXT	4	2 (1.9)
CTX/ CAZ/CIP/LVX/Te/AM	4	1 (0.9)
CTX/CAZ/Te/AM/SXT	4	14 (13.2)
CTX/CIP/LVX/AM/SXT	4	2 (1.9)
CIP/LVX/Te/AM/SXT	4	4 (3.8)
CTX/Te/AM/SXT	4	12 (11.3)
CIP/Te/AN/SXT	4	1 (0.9)
CTX/CAZ/CIP/LVX/AM	3	1 (0.9)
CTX/CAZ/AM/SXT	3	6 (5.7)
CTX/CAZ/TZP/AM	3	1 (0.9)
CTX//CIP/LVX/AM	3	1 (0.9)
CIP/LVX/Te/SXT	3	1 (0.9)
CTX/Te/AM	3	4 (3.8)
CTX/AM/SXT	3	2 (1.9)
CIP/Te/AM	3	1 (0.9)
CIP/AM/SXT	3	1 (0.9)
LVX/Te/SXT	3	1 (0.9)
Te/AM/SXT	3	24 (22.7)

*AM* ampicillin, *AN* amikacin, *CAZ* ceftazidime, *CIP* ciprofloxacin, *CTX* cefotaxime, *LVX* levofloxacin, *STX* trimethoprim + sulfamethoxazole, *Te* tetracyclines, *TZP* piperacillin + tazobactam, *MDR* multidrug resistant

### Antibiotic resistance genes

Four genes responsible for β-lactamase production have been investigated by PCR from 71 ESBL positive phenotypes. The results showed 67 (94.4%) isolates contained at least 1 of these ESBL encoding genes. The *blaTEM* gene was more common with frequency of 60 (84.5%), followed by *blaCTX-M9* gene 49 (69.0%), *blaSHV* gene 35 (49.3%), and *blaOXA* gene 28 (39.4%). Molecular evaluation of ESBL-producing isolates indicates 4, 3, 2 and 1 gene patterns (4.2%, 16.9%, 42.3% and 31.0%, respectively). The highest co-existence rate of β-lactamase producing genes belonged to *blaCTX-M9/blaTEM*, followed by *blaOXA/blaTEM*, *blaCTX-M9/blaSHV, blaCTX-M9/blaOXA* and *blaSHV/blaTEM* (31.0%, 23.9%, 19.7%, 16.9% and 16.9%, respectively). The Molecular Patterns are demonstrated in Table 5.

**Table 5:** Molecular pattern of 71 ESBL positive strains

Molecular Pattern	No. (%)
CTX-M9/SHV/OXA/TEM	3 (4.2)
CTX-M9/SHV/OXA	2 (2.8)
CTX-M9/SHV/TEM	3 (4.2)
CTX-M9/OXA/TEM	6 (8.5)
SHV/OXA/TEM	1 (1.4)
CTX-M9/SHV	6 (8.5)
CTX-M9/OXA	1 (1.4)
CTX-M9/TEM	10 (14.1)
SHV/TEM	6 (8.5)
OXA/TEM	7 (9.9)
CTX-M9	4 (5.6)
SHV	4 (5.6)
OXA	1 (1.4)
TEM	13 (18.3)
None	4 (5.6)

*ESBL* extended spectrum β-lactamase

### Phylogenetic analysis

**Fig. 1:**
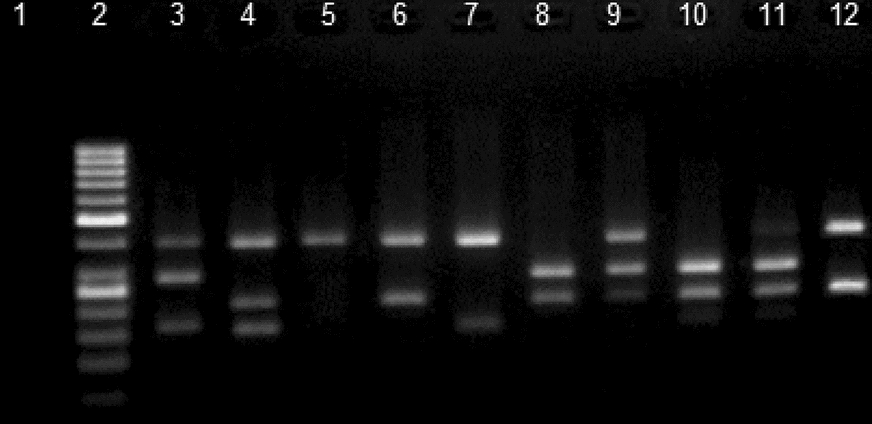
Phylo-typing of STEC strains. *arpA* (400 bp), *chuA* (288 bp), *yjaA* (211 bp) and TspE4.C2 (152 bp). Lane 1, negative control; lane 2, molecular weight marker (50 bp, Fermentas); lane 3, group D (+ + − +); lane 4, unknown (+ − + +); lane 5, group A (+ − − −); lanes 6&12, group C (+ − + −); lane 7, group B1 (+ − − +); lanes 8&10, group B2 (− + + −) and lanes 9&11, group E (+ + + −)

**Fig. 2:**
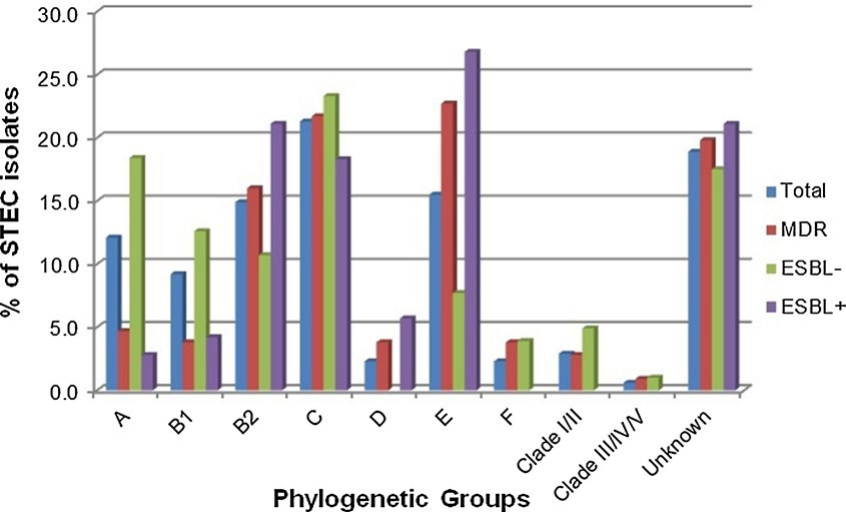
Distribution of Phylogenetic Groups among STEC isolates. *STEC* Shiga toxin‐producing *Escherichia coli*, *MDR* multidrug resistant, *ESBL* extended spectrum β-lactamase

The 174 STEC isolates were assigned into different phylogroups according to Clermont’s quadruplex scheme (Fig. 1). The phylotyping results revealed Group C as the most frequent Group (21.3% occurrence), followed by Groups E (15.5%), B2 (14.9%), A (12.1%) and B1 (9.2%). The prevalence of Groups D and F was 2.3%; moreover approximately 18.9% of the isolates remained unclassified. The MDR and ESBL producing isolates represented more affiliation with Group E (Fig. 2).

### MLST analysis

According to virulence genes, ESBL genes and resistance profile, total of 25 isolates; 18 STEC and 7 EPEC (previously published data), were analyzed using Pasteur MLST scheme. Examining gene sequences of the strains and assigning specific alleles for each locus resulted in the identification of diverse sequence types (STs) (Table 6). The tested isolates exhibited 21 sequence types among which the most frequent ones were ST506 (3 isolates), ST77 (2 isolates) and ST1007 (2 isolates). ST506 were represented by ESBL producers.

**Table 6:** Characteristics of diarrheagenic *Escherichia coli* strains and their affiliation to sequence types

Isolate	PG	Virulence gene	Resistance profile	β-lactamase gene	PST	Allelic profile^b^
B1013053	clade V	stx1	Te, AM, SXT	–	1005^a^	152,189,7,132,162,127,130,77
B2011071	F	stx1	CIP,LVX,Te, AM, SXT	–	1011^a^	13,148,194,16,12,25,90,19
E3012101	E	stx1	ESBL+, CTX,Te, AM,SXT	CTX,SHV,TEM	1012^a^	10,33,18,166,5,28,2,2
E3021041	A	stx1	ESBL+, CTX,CAZ,Te, AM,SXT	SHV,TEM	353	11,3,4,3,15,1,4,40
F4011011	A	stx1	Te, AM, SXT	–	1006^a^	8,2,7,3,186,1,4,2
H1012052	A	stx1,stx2	ESBL+, CTX,Te, AM,SXT	SHV,TEM	83	11,3,4,3,15,1,4,16
H2012086	A	stx1,stx2,eae	ESBL+, CTX,Te, AM,SXT	SHV,TEM	191	8,2,7,3,61,1,4,2
H2022075	B2	stx1	ESBL+, CTX,CAZ,CIP,LVX,Te, AM, SXT	SHV,OXA,TEM	43	9,1,15,7,4,9,6,9
H4022014	B2	stx1	sensitive	–	53	1,7,1,9,20,20,1,6
H4022024	E	eae	ESBL+, CTX,CAZ,Te, AM,SXT	SHV,TEM	1013^a^	10,2,149,17,18,116,16,40
I2021071	B2	stx1	ESBL+, CTX,Te, AM,SXT	CTX,TEM	506	9,134,74,134,4,72,1,9
I2022061	B1	stx1	ESBL+, CTX,CAZ,Te, AM,SXT	CTX,TEM	186	7,33,18,68,5,8,2,2
J2022082	B1	stx1,eae	sensitive	–	1007^a^	25,3,4,146,78,29,2,5
J2023073	A	stx1,eae	ESBL+, CTX,Te, AM,SXT	OXA,TEM	446	10,2,3,3,7,1,4,2
J4011022	D	stx1,eae	ESBL+, CTX,CAZ,Te, AM,SXT	OXA,TEM	3	3,8,5,11,8,3,5,3
J4011121	B1	stx2	CTX,Te,AM	–	1007^a^	25,3,4,146,78,29,2,5
J4021026	B1	eae	Te, AM, SXT	–	1009^a^	5,3,4,52,84,1,16,66
K3012111	B2	stx1	ESBL+, CTX,CAZ,TZP,AM	CTX,SHV,OXA,TEM	506	9,134,74,134,4,72,1,9
K4012127	A	eae	AM, SXT	–	367	8,104,7,3,7,1,4,2
L3013091	D	stx1,stx2	ESBL+, CTX,Te, AM	NONE	1008^a^	3,8,5,11,161,3,5,3
N1022052	D	eae	ESBL+, CTX, AM	CTX,SHV,TEM	77	3,43,31,33,8,3,5,11
N1022054	B2	eae	ESBL+, CTX,CAZ,Te, AM,SXT	CTX,SHV,OXA,TEM	506	9,134,74,134,4,72,1,9
N3013101	B1	stx1	Te, AM, SXT	–	636	7,3,3,68,74,7,4,5
O2021062	A	eae	ESBL+, CTX,CAZ,TZP,CIP,LVX,Te, AM,SXT	CTX,SHV,TEM	1014^a^	10,2,3,163,18,116,16,22
O4011022	D	eae	Te, AM, SXT	–	77	3,43,31,33,8,3,5,11

*PG* polygenetic group, *ESBL* extended spectrum β-lactamase, *PST* pasteur sequence type

^a^New; ^b^Allelic profile based on MLST of 8 housekeeping genes (dinB, icdA, pabB, polB, putP, trpA, trpB,and uidA)

## Discussion

Of the 340 *E. coli* isolates in this study, 174 strains were identified as STEC among which prevalence of *stx1* and *stx2* genes was 98.3% and 5.7%, respectively. Although in general infection of humans by *stx2a* may cause more severe clinical outcomes [2, 21], a recent study in England indicated that presence of stx1a can also be associated with severe disease [21]. The current study provides results of antibacterial assessment; molecular evaluation of ESBL-producing isolates and phylogenetic groups of STEC strains associated with diarrheal patients in Iran. The patients comprised of 55.7% males and 44.3% females distributed among all age groups with higher incident in ≤ 6 years, which was similar to findings of other studies [22, 23].

Overuse of antibiotics with and/or without prescription is responsible for emergence of multidrug resistance among clinical isolates, which is a serious public health issue and can create therapeutic difficulties for patients. The findings of this study show high levels of resistance to ampicillin, trimethoprim–sulfamethoxazole and tetracycline among STEC, which is in agreement with previous findings in Japan, China, South Africa, Iran, Mexico and Michigan [24–29]. Also relatively lower levels of resistance to cefotaxime, ceftazidime, ciprofloxacin and levofloxacin was revealed, a finding which was similar to those reported by others [30, 31], and opposite to that reported by Zhang et al. in china [25]. Among antibiotics tested, highest sensitivity was toward imipenem and ertapenem with 99.4%, followed by amikacin and piperacillin–tazobactam with 96.0% and 93.1%, respectively. Similar pattern was reported from India [30]. The prevalence of MDR was 60.9%, which was both lower [25] and higher [24] than results reported by others. Furthermore, almost all of the ESBL-producing STEC strains were MDR.

The presence of ESBL-producing isolates in clinical samples is worrisome in both developed and developing countries. Rapid expansion of ESBL positive isolates highly affects the activity of broad-spectrum antibiotics, creating major therapeutic difficulties [32]. Also, the involvement of horizontal gene transfer in the spread of resistance determinants and the fact that ESBL encoding plasmids also carry resistance genes for other antimicrobial drugs poses a serious challenge. Therefore, the detection of gene variants in β-lactamase-producing bacteria is essential information for the appropriate and effective treatment of patients. In the current study, the 71 ESBL-producing STEC isolates were analyzed by PCR for the presence of 4 β-lactamase genes. The *blaTEM* was more common, followed by *blaCTX-M9*, *blaSHV* and *blaOXA.* In respect to higher prevalence of *blaTEM,* our findings are similar with a study from India [33], whereas in other studies *blaCTX-M* was more common [30, 31].

Phylogenetic analysis of *E. coli* clinical strains provides information about the frequency of occurrence in the environment. In this study, we determined the phylogenetic group of 174 STEC strains isolated from the stool of diarrheal patients and showed that the majority of STEC strains belonged to 1 of 5 phylogroups; C, E, B2, A and B1, while ESBL positive and MDR strains represented more affiliation with Group E. Furlan et al. [34] assessed the prevalence of phylogenetic groups in STEC isolated from sheep and revealed that group E was most prevalent, followed by B1, A and B2. Jajarmi et al. [35] reported that phylogenetic group B1 then A were the most prevalent in STEC strains isolated from goats. Phylogenetic groups A and B2 were also reported by Ahumada-Santos et al. as the most frequent phylogroups among children with diarrheagenic *E. coli* [36]. These differences in distribution of phylogenetic groups in different studies may be due to varying conditions in sampling areas.

In current study, we also used Pasteur MLST scheme to analyze the diarrheagenic *E. coli* population structure using the sequences of eight housekeeping genes, and MLST results indicated a highly diverse sequence types (STs), which are in accordance with other findings [37, 38].

## Conclusion

*Escherichia coli* isolates in this study have demonstrated high levels of resistance to various antimicrobial agents. The emergences of ESBL-producing isolates were detected, which poses a serious public health challenges. Our results also revealed worrying increase in the prevalence of MDR among ESBL-producing STEC, especially those affiliated to phylogroup E. These findings emphasize the health risks and difficulties that could be encountered in eradicating STEC infections. Thus more research should be directed towards periodical surveillance studies to screen/identify patients that are carriers of ESBL-producing bacteria, in order to develop an effective antibiotic therapy guideline. Moreover, to prevent the further spread of MDR isolates, empirical treatments and overuse of antibiotics by patients should be discouraged.

## Data Availability

The datasets used and/or analyzed during the current study are available from the corresponding author on reasonable request.

## Abbreviations

STEC: Shiga toxin‐producing *Escherichia coli*
stx: Shiga toxin
HC: Hemorrhagic colitis
HUS: Hemolytic uremic syndrome
ESBL: Extended-spectrum β-lactamase
MLST: Multi-locus sequence types
PCR: Polymerase chain reaction
MDR: Multidrug resistance
*eae*: Intimin
CLSI: Clinical and laboratory standards institute
ST: Sequence type
EPEC: Enteropathogenic *Escherichia coli*

## References

[1] Gyles C (2007). Shiga toxin-producing *Escherichia coli*: an overview. J Anim Sci.

[2] Smith JL, Fratamico PM, Gunther IVNW (2014). Shiga toxin-producing *Escherichia coli*. Advances in applied microbiology.

[3] Mir RA, Kudva IT (2019). Antibiotic-resistant Shiga toxin-producing *Escherichia coli*: an overview of prevalence and intervention strategies. Zoonoses Public Health.

[4] Erb A, Stürmer T, Marre R, Brenner H (2007). Prevalence of antibiotic resistance in *Escherichia coli*: overview of geographical, temporal, and methodological variations. Eur J Clin Microbiol Infect Dis.

[5] Babic M, Hujer AM, Bonomo RA (2006). What's new in antibiotic resistance? Focus on beta-lactamases. Drug Resist Updates.

[6] Bush K, Bradford PA (2020). Epidemiology of β-lactamase-producing pathogens. Clin Microbiol Rev.

[7] Alves H, de Cruz F, de Assis P, Pessoa JD, Trevelin L, de Leal A (2017). Antibiotic resistance among *Escherichia coli*: isolates and novel approaches to the control of *E. coli* infections. Recent advances on physiology, pathogenesis and biotechnological applications.

[8] Clermont O, Christenson JK, Denamur E, Gordon DM (2013). The C lermont E scherichia coli phylo-typing method revisited: improvement of specificity and detection of new phylo-groups. Environ Microbiol Rep.

[9] Reid CJ, Wyrsch ER, Chowdhury PR, Zingali T, Liu M, Darling AE (2017). Porcine commensal *Escherichia coli*: a reservoir for class 1 integrons associated with IS26. Microbial genomics.

[10] Nowrouzian FL, Clermont O, Edin M, Östblom A, Denamur E, Wold AE (2019). *Escherichia coli* B2 phylogenetic subgroups in the infant gut microbiota: predominance of uropathogenic lineages in swedish infants and enteropathogenic lineages in Pakistani Infants. Appl Environ Microbiol.

[11] Mosquito S, Pons MJ, Riveros M, Ruiz J, Ochoa TJ (2015). Diarrheagenic *Escherichia col*i phylogroups are associated with antibiotic resistance and duration of diarrheal episode. Sci World J.

[12] Garcia LS (2010). Clinical microbiology procedures handbook.

[13] Müller D, Greune L, Heusipp G, Karch H, Fruth A, Tschäpe H (2007). Identification of unconventional intestinal pathogenic *Escherichia coli* isolates expressing intermediate virulence factor profiles by using a novel single-step multiplex PCR. Appl Environ Microbiol.

[14] Carneiro L, Lins M, Garcia F, Silva A, Mauller P, Alves G (2006). Phenotypic and genotypic characterisation of *Escherichia coli* strains serogrouped as enteropathogenic *E. coli* (EPEC) isolated from pasteurised milk. Int J Food Microbiol.

[15] Wayne P (2016). Performance standards for antimicrobial susceptibility testing, CLSI supplement M100S.

[16] Basak S, Singh P, Rajurkar M (2016). Multidrug resistant and extensively drug resistant bacteria: a study. J Pathog.

[17] Navon-Venezia S, Chmelnitsky I, Leavitt A, Carmeli Y (2008). Dissemination of the CTX-M-25 family β-lactamases among Klebsiella pneumoniae, *Escherichia coli* and *Enterobacter cloacae* and identification of the novel enzyme CTX-M-41 in *Proteus mirabilis* in Israel. J Antimicrob Chemother.

[18] Van TTH, Chin J, Chapman T, Tran LT, Coloe PJ (2008). Safety of raw meat and shellfish in Vietnam: an analysis of *Escherichia coli* isolations for antibiotic resistance and virulence genes. Int J Food Microbiol.

[19] Gheorghe I, Czobor I, Chifiriuc MC, Borcan E, Ghiţă C, Banu O (2014). Molecular screening of carbapenemase-producing Gram-negative strains in Romanian intensive care units during a one year survey. J Med Microbiol.

[20] Maynou G, Migura-Garcia L, Chester-Jones H, Ziegler D, Bach A, Terré M (2017). Effects of feeding pasteurized waste milk to dairy calves on phenotypes and genotypes of antimicrobial resistance in fecal *Escherichia coli* isolates before and after weaning. J Dairy Sci.

[21] Byrne L, Adams N, Jenkins C (2020). Association between Shiga Toxin-producing *Escherichia coli* O157: H7 stx gene subtype and disease Severity, England, 2009–2019. Emerg Infect Dis.

[22] Majowicz SE, Scallan E, Jones-Bitton A, Sargeant JM, Stapleton J, Angulo FJ (2014). Global incidence of human Shiga toxin–producing *Escherichia coli* infections and deaths: a systematic review and knowledge synthesis. Foodborne Pathog Dis.

[23] Käppeli U, Hächler H, Giezendanner N, Beutin L, Stephan R (2011). Human infections with non-O157 Shiga toxin–producing *Escherichia coli*, Switzerland, 2000–2009. Emerg Infect Dis.

[24] Kubomura A, Sekizuka T, Onozuka D, Murakami K, Kimura H, Sakaguchi M (2020). Truncated class 1 integron gene cassette arrays contribute to antimicrobial resistance of diarrheagenic *Escherichia coli*. Biomed Res Int.

[25] Zhang S-X, Zhou Y-M, Tian L-G, Chen J-X, Tinoco-Torres R, Serrano E (2018). Antibiotic resistance and molecular characterization of diarrheagenic *Escherichia coli* and non-typhoidal *Salmonella* strains isolated from infections in Southwest China. Infect Dis Poverty.

[26] Omolajaiye S, Afolabi K, Iweriebor B (2020). Pathotyping and antibiotic resistance profiling of *Escherichia coli* isolates from children with acute diarrhea in amatole district municipality of Eastern Cape, South Africa. BioMed Res Int.

[27] Abbasi E, Mondanizadeh M, van Belkum A, Ghaznavi-Rad E (2020). Multi-drug-resistant diarrheagenic *Escherichia coli* pathotypes in pediatric patients with gastroenteritis from central Iran. Infect Drug Resist.

[28] Amézquita-López BA, Quiñones B, Soto-Beltrán M, Lee BG, Yambao JC, Lugo-Melchor OY (2016). Antimicrobial resistance profiles of Shiga toxin-producing *Escherichia coli* O157 and Non-O157 recovered from domestic farm animals in rural communities in Northwestern Mexico. Antimicrob Resist Infect Control.

[29] Mukherjee S, Mosci RE, Anderson CM, Snyder BA, Collins J, Rudrik JT (2017). Antimicrobial drug–resistant Shiga toxin–producing *Escherichia coli* infections, Michigan, USA. Emerg Infect Dis.

[30] Mandal A, Sengupta A, Kumar A, Singh UK, Jaiswal AK, Das P (2017). Molecular epidemiology of extended-spectrum β-lactamase–producing *Escherichia coli* pathotypes in diarrheal children from low socioeconomic status communities in Bihar, India: emergence of the CTX-M Type. Infect Dis Res Treat.

[31] Haghighatpanah M, Nejad ASM, Mojtahedi A, Amirmozafari N, Zeighami H (2016). Detection of extended-spectrum β-lactamase (ESBL) and plasmid-borne blaCTX-M and blaTEM genes among clinical strains of *Escherichia coli* isolated from patients in the north of Iran. J Glob Antimicrob Resist.

[32] Adler A, Katz DE, Marchaim D (2016). The continuing plague of extended-spectrum β-lactamase–producing Enterobacteriaceae infections. Infect Dis Clin.

[33] Singh T, Singh PK, Das S, Wani S, Jawed A, Dar SA (2019). Transcriptome analysis of beta-lactamase genes in diarrheagenic *Escherichia coli*. Sci Rep.

[34] Furlan JPR, Gallo IFL, de Campos ACLP, Navarro A, Kobayashi RKT, Nakazato G (2019). Characterization of non-O157 Shiga toxin-producing *Escherichia coli* (STEC) obtained from feces of sheep in Brazil. World J Microbiol Biotechnol.

[35] Jajarmi M, Askari Badouei M, Ghanbarpour R, Karmostaji A, Alizade H (2019). Antimicrobial resistance patterns and phylogenetic analysis of Shiga toxin-producing *Escherichia coli* strains from goats using both clermont phylogenetic schemes. Bulgarian J Vet Med.

[36] Ahumada-Santos YP, Báez-Flores ME, Díaz-Camacho SP, de Jesús U-B, Eslava-Campos CA, Parra-Unda JR (2020). Association of phylogenetic distribution and presence of integrons with multidrug resistance in *Escherichia coli* clinical isolates from children with diarrhoea. J Infect Public Health.

[37] Cadona JS, Bustamante AV, González J, Sanso AM (2016). Genetic relatedness and novel sequence types of Non-O157 Shiga toxin-producing *Escherichia coli* strains isolated in Argentina. Front Cell Infect Microbiol.

[38] Fierz L, Cernela N, Hauser E, Nüesch-Inderbinen M, Stephan R (2017). Human infections with Shiga toxin-producing *Escherichia coli*, Switzerland, 2010–2014. Front Microbiol.

